# Antianemic Treatment of Cancer Patients in German Routine Practice: Data from a Prospective Cohort Study—The Tumor Anemia Registry

**DOI:** 10.1155/2016/8057650

**Published:** 2016-02-04

**Authors:** Tilman Steinmetz, Jan Schröder, Margarete Plath, Hartmut Link, Michèle Vogt, Melanie Frank, Norbert Marschner

**Affiliations:** ^1^Outpatient Clinic for Hematology and Oncology, Sachsenring 69, 50677 Cologne, Germany; ^2^Outpatient Clinic for Oncology, Kettwiger Strasse 62, 45468 Mülheim an der Ruhr, Germany; ^3^Outpatient Clinic for Oncology, Prinzregentenstrasse 1, 86150 Augsburg, Germany; ^4^Department for Internal Medicine I, Westpfalz-Klinikum, Hellmut-Hartert-Strasse 1, 67655 Kaiserslautern, Germany; ^5^iOMEDICO, Hanferstrasse 28, 79108 Freiburg, Germany; ^6^Outpatient Clinic for Interdisciplinary Oncology and Hematology, Wirthstrasse 11c, 79110 Freiburg, Germany

## Abstract

The aim of this prospective cohort study was to assess current antianemic treatment of cancer patients in German routine practice, including diagnostics, treatments, and quality of life (QoL). 88 study sites recruited 1018 patients at the start of antianemic treatment with hemoglobin (Hb) levels <11 g/dL (females) or <12 g/dL (males). Patients were followed up for 12 weeks. 63% of the patients had inoperable solid tumors, 22% operable solid tumors, and 15% hematological malignancies. Over 85% received chemotherapy. Median age was 67 years; 48% were male. Red blood cell transfusions (RBCTx) were given to 59% of all patients and to 55% of the patients with Hb ≥8 g/dL on day 1 of the observation period (day 1 treatment). Erythropoiesis-stimulating agents (ESAs) were the second most frequently applied day 1 treatment (20%), followed by intravenous (IV) iron (15%) and ESA + IV iron (6%). Only about a third of patients were tested for blood serum iron parameters at the start of treatment. Overall, more than half of the patients had long-term responses to antianemic therapy. Our data suggest that in routine practice diagnostics for treatable causes of anemia are underused. A high proportion of cancer patients receive RBCTx. It should be discussed whether thorough diagnostics and earlier intervention could decrease the need for RBCTx. This trial is registered with NCT01795690.

## 1. Introduction

Anemia is defined as a hemoglobin (Hb) level of <12 g/dL for nonpregnant women and <13 g/dL for men, according to the World Health Organization [1]. It is a common complication of multifactorial etiology among patients with malignant diseases. The European Cancer Anemia Survey (ECAS) reported an overall anemia incidence (Hb <12 g/dL) of more than 50% during the 6-month survey period for patients with solid or hematological tumors who received their first anticancer treatment. Anemia incidence was almost 65% in patients receiving chemotherapy [2]. Low Hb levels are associated with poor physical performance status [2–5] and decreased quality of life (QoL) [3, 6–9], indicating a need for early antianemic treatment.

Treatment strategies include red blood cell transfusions (RBCTx), erythropoiesis-stimulating agents (ESAs), and iron supplementation either alone or in combination with ESAs. Treatment decision-making should be based on the best benefit-to-risk ratio for each patient and depends on patients' Hb level, the presence of symptoms, and the underlying cause for anemia as evaluated by blood parameters such as ferritin, transferrin saturation (TSAT), folate, and vitamin B12 [10–12]. While the National Comprehensive Cancer Network (NCCN) has published a comprehensive guideline on anemia management, current guidelines in Europe focus on the application of ESAs and/or transfusions. The administration of intravenous (IV) iron is the treatment of choice for cancer patients with anemia due to absolute iron deficiency (AID). It has been shown to improve efficacy and is thus recommended in combination with ESAs for patients with functional iron deficiency (FID) [10–12]. If iron deficiency is excluded, the European Organization for Research and Treatment of Cancer (EORTC) recommends ESAs to treat symptomatic anemia with Hb levels ≥9 g/dL and to assess whether transfusions are required in case of Hb levels <9 g/dL [10]. The NCCN advises thorough diagnostics for possible causes of anemia and subsequent treatment of these. If no treatable cause can be identified, transfusions are recommended depending on the presence of symptoms and comorbidities. ESAs are suggested for anemic patients undergoing palliative cancer treatment but not for patients receiving chemotherapy with curative intent [12]. The German guideline on the use of transfusions considers them an option depending on severity and symptoms of anemia, especially when rapid, short-term improvement of Hb levels <8 g/dL is required [13].

Prospective, observational studies can be used to assess the current state of care. In 2001/2002, the ECAS assessed prevalence, incidence, and treatment of anemia in more than 15,000 cancer patients in Europe. Over all patients, ESA therapy was the most frequently used antianemic treatment, while transfusions were most commonly applied in anemic patients with Hb levels ≤9.9 g/dL receiving chemotherapy [2]. In 2004/2005, the German Cancer Anemia Registry (CAR) was a survey on the planned anemia management of almost 2,000 cancer patients in German routine care. Overall, the three predefined treatment strategies “to correct underlying disorder causative of anemia” (e.g., iron or vitamin deficiency or bone marrow infiltration), “to use transfusions as first-line treatment,” and “to use ESA as first-line treatment” were selected equally frequently, while diagnostic measures were used in two-thirds of patients only [3].

Here, we present data on the current anemia management in cancer patients from the Clinical Tumor Anemia Registry (TAR) conducted in 2012/2013. This paper addresses the treatment reality of patients with cancer and/or therapy related anemia, the use of diagnostic measures, and effectiveness of treatment based on changes in Hb values and QoL within three months after the start of antianemic treatment.

## 2. Patients and Methods

### 2.1. Study Design

The TAR was an open, prospective, multicenter, longitudinal, observational study investigating the treatment reality of patients with cancer-induced anemia in Germany. It was conducted according to the Declaration of Helsinki, reviewed by an ethics committee, and registered in the ClinicalTrials.gov registry (NCT01795690).

### 2.2. Patients

Eligible patients were ≥18 years old, with diagnosed cancer, irrespective of tumor type, and about to start antianemic therapy with baseline Hb levels <11 g/dL (females) or <12 g/dL (males). Antianemic treatment was started no longer than 7 days prior to signing written informed consent. Additional inclusion criteria comprised an Eastern Cooperative Oncology Group performance status of 0–3 and life expectancy of >16 weeks. Patients with myelodysplastic syndrome or an experimental antianemic therapy as part of a clinical trial were excluded. Study sites were encouraged to enroll patients consecutively to ensure unselected recruitment. Patients were treated according to physicians' choice based on patients' individual needs.

### 2.3. Data Collection

At the time of enrolment, data on patients' sociodemographics, tumor entity, type of antineoplastic treatment, concomitant diseases, previous antianemic treatments, and current laboratory parameters were documented. Comorbidity was assessed using the Charlson Comorbidity Index [16]. During the 12-week observational period, antianemic treatment and laboratory parameters were documented. Data were collected from patients' medical files and transferred to a secure web-based electronic case report form (eCRF) by physicians or trained study nurses. Implemented automatic completeness and plausibility checks, and if necessary direct contact with the study site, were done for quality assurance. To determine QoL, patients completed the Functional Assessment of Cancer Therapy Anemia (FACT-An) questionnaire at enrolment and 6 and 12 weeks later. The initial questionnaire was filled at the study site; the remaining two were mailed to the patients, filled at home, and returned by mail in prepaid envelopes. All patients who returned the baseline questionnaire were included in the analysis of patient-reported outcomes.

### 2.4. Patient Cohort and Statistical Analysis

Of all patients recruited, those with documented baseline Hb (measured no longer than 7 days before the start of antianemic treatment) were eligible for the final analysis. Patients who received one of the four standard antianemic treatments (RBCTx, ESA, IV iron, or ESA + IV iron) on day 1 of the observation period (day 1 treatment) were included in the present analysis. Patients were categorized by (1) their type of disease (solid operable tumor/potentially curative, solid inoperable tumor/palliative intention, and hematological tumor) and by (2) their day 1 treatment. The frequency of diagnostic measurements at the start of treatment was calculated. For this purpose, the number of patients for whom specified blood parameters were measured at least once within 4 weeks until 2 weeks after the beginning of antianemic treatment was determined. To analyze the effectiveness of treatments, the proportion of “responders” and ΔHb(final) and ΔHb(max) were determined. ΔHb(final) was defined as the difference between the baseline Hb and the last Hb documented within the observation period, but at least 4 weeks after the start of treatment. ΔHb(max) was defined as the difference between the baseline Hb and the highest Hb documented. “Responders” were all patients with final Hb of >11 g/dL or with ΔHb(final) of ≥1.5 g/dL, with the final Hb being the last documented Hb within the observation period, but at least 4 weeks after the start of antianemic treatment.

The FACT-An total score and the anemia-specific subscale score were determined according to the questionnaire's manual. Missing data within a questionnaire were handled according to the questionnaire's manual [17]. Median scores were calculated for each time point and patient sample. No imputations for missing questionnaires were performed. Improvements of seven points on the FACT-An total scale and four points on the anemia subscale were considered clinically meaningful [14, 15]. The statistical analysis was performed using STATISTICA (StatSoft, Inc.) version 10.0, R version 2.15.1, and IBM SPSS Statistics version 19.0.

## 3. Results and Discussion

### 3.1. Patients' Characteristics and Day 1 Treatment

**Figure 1:**
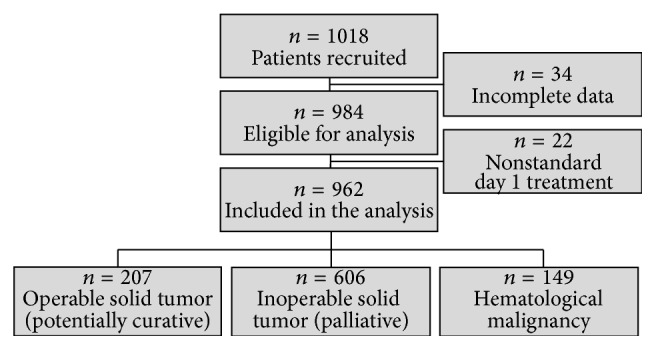
Patient recruitment, patient cohort, and type of disease.

Between March 2012 and September 2013, 216 office-based medical oncologists from 88 study sites recruited 1018 patients. Of these patients, 984 were eligible for analysis. 22 patients were excluded because treatment sample sizes were too small for meaningful analysis. They received nonstandard day 1 treatments (8 oral iron, 5 ESA + RBCTx, 4 oral iron + RBCTx, 2 oral iron + IV iron + RBCTx, 2 IV iron + RBCTx, and 1 oral iron + IV iron), and to this end 962 patients were included in the present study (Figure 1).

Table 1 displays the baseline sociodemographic and clinical characteristics. Overall, 85% of the patients (*n* = 813) had solid tumors, predominantly breast, colorectal, and non-small cell lung cancer (NSCLC), whereas the remaining patients (15%, *n* = 149) were affected by hematological malignancies. 75% of the solid tumors were inoperable (palliative patients, *n* = 606). Mean baseline Hb was 8.9 g/dL.

**Figure 2:**
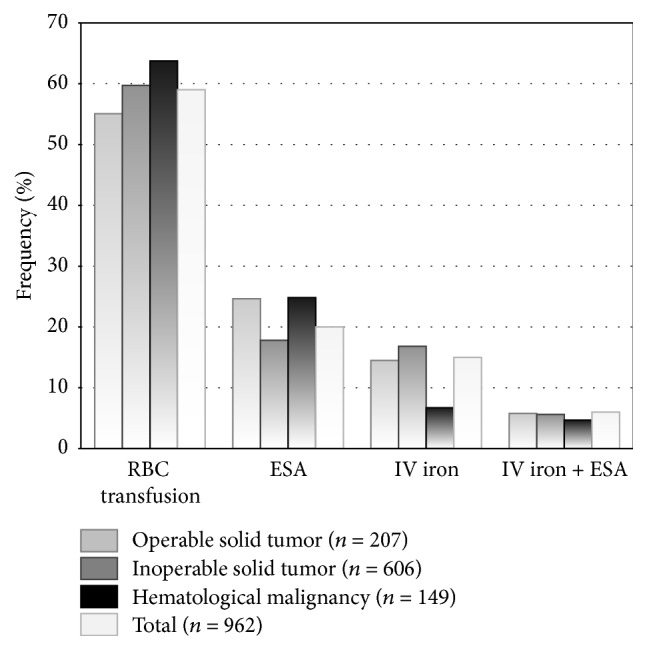
Frequency of antianemic day 1 treatments by type of disease.

The majority of patients (88%, *n* = 850) received chemotherapy, of which about half were platinum based (Table 1).  Figure 2 presents the frequency of the most common day 1 treatments according to type of disease (*n* = 962). Overall, 59% (*n* = 571) of the patients received RBCTx, 20% (*n* = 196) underwent ESA therapy, and 15% (*n* = 142) were treated with IV iron. A combination of ESAs and IV iron was the treatment of choice for the minority of patients (6%, *n* = 53) (Table 1). Thus, approximately 40% of the patients received antianemic therapy with ESA, IV iron, or ESA + IV iron.

Patients with inoperable solid tumors and patients with hematological malignancies were treated more often with RBCTx (60% and 64%, resp.) than patients with operable solid tumors (55%). ESA therapy was used less frequently in patients with inoperable solid tumors than in patients with operable solid tumors and hematological malignancies (18% versus 25%). Of all patients with solid tumors receiving ESAs, patients with breast cancer constitute approximately one-third. 20–30% of the patients with solid tumors and treated with IV iron had colorectal cancer (Table 1).

Approximately 20% of the patients had received previous antianemic therapies within 4 weeks before day 1 of the observation period, mostly RBCTx (data on file).

Our data show that in 2012/2013 transfusions accounted for almost 60% of day 1 antianemic treatments in German routine practice, while ESA (alone or with IV iron) was used in 26% and IV iron alone in 15% of patients. In 2004/2005, the German CAR study reported that transfusions were planned as “first-line” antianemic therapy for almost 35% of anemic cancer patients, whereas ESAs were chosen for 39% and strategies “correcting the underlying disorder” for 26% of patients [3]. Mean Hb for requiring treatment was 9.4 g/dL in CAR, while mean Hb at the start of treatment was 8.9 g/dL in TAR. Mean Hb triggering transfusion was 8.7 g/dL in CAR, while mean Hb at the start of transfusion as day 1 treatment was 8.6 g/dL in TAR. Mean Hb when ESAs, IV iron, and ESA + IV iron were chosen was 9.7 g/dL in CAR and between 9 and 10 g/dL in TAR (data on file).

In 2001/2002, the ECAS reported that approximately 38% of those patients receiving any antianemic therapy were treated with transfusions (alone or in combination with iron) at any time during the observational period, while approximately 45% received ESAs (alone or in combination with iron and/or transfusion) [2].

It has to be noted that CAR also included patients with Hb levels <12 g/dL (females) or <13 g/dL (males), who are less likely to receive transfusions. On the other hand, 33% of patients in CAR had a lymphoproliferative malignancy compared to 15% in TAR, with these patients being more likely to receive transfusions in both data sets. The ECAS recruited all patients, independently of anemia, and also anemic patients that did not require treatment. Mean Hb at the start of treatment was higher in ECAS (9.7 g/dL) than in TAR (8.9 g/dL). Patients undergoing chemotherapy and whose Hb was <9 g/dL at the start of antianemic treatment were treated most frequently with transfusions (53%). In contrast, 71% of TAR patients receiving transfusions had Hb of <9 g/dL.

In summary, our data indicate that the use of transfusions as antianemic treatment might have increased in the last decade, while the use of ESA has decreased. Due to the limitations of historic controls, it cannot be excluded that the differences seen in the frequencies of treatments in ECAS, CAR, and TAR are at least partially caused by differences in the design of these studies (inclusion criteria) resulting in different patients recruited (e.g., with lower Hb in TAR) and thus receiving different treatments (e.g., more transfusions in TAR).

### 3.2. RBC Transfusions in Patients with Hb Levels ≥8 g/dL

In general, patients receiving RBCTx as day 1 treatment showed lower baseline Hb values than patients receiving other antianemic therapies (Table 1).

Overall, 85% (*n* = 813) of all patients had baseline Hb levels ≥8 g/dL. Of these patients, 55% (*n* = 443) received RBCTx. This also means that, of all patients receiving transfusions (*n* = 571), almost 80% (*n* = 443) had baseline Hb values ≥8 g/dL. Study sites reported the presence of anemic symptoms for 88% of patients with Hb levels ≥8 g/dL. In total, 71% (*n* = 406) of the patients who received transfusions (*n* = 571) had Hb values <9.0 g/dL at the start of treatment (data on file).

The high rates of RBCTx, especially in patients with Hb ≥8 g/dL in TAR, are concerning, considering the various risks, such as transfusion-transmitted infections, transfusion-related circulatory overload, iron overload, anaphylactic reactions, and thromboembolism [8, 12, 18]. While RBCTx are the only option when immediate correction of anemic symptoms is required, there is ongoing debate about the Hb that should trigger transfusions, which is reflected in several changes in guidelines over time [18–20]. According to the EORTC guideline on the use of ESAs, patients should be evaluated for the need of transfusions if their Hb level is <9 g/dL [21]. Guidelines of the German Medical Association indicate the use of transfusions for patients with symptomatic anemia whose Hb level is <8 g/dL and/or for patients with symptomatic cardiovascular disease and the additional presence of physiologic transfusion triggers, such as tachycardia or hypotension, along with an Hb level between 8 and 10 g/dL [13]. The high rate of transfusions in patients with Hb levels ≥8 g/dL in TAR may only partly be explained by the presence of physiologic transfusion triggers, data on which were not collected within this study. There may be other rationales for applying transfusions more often than other treatments in patients with Hb levels ≥8 g/dL and in the study cohort as a whole.

### 3.3. Testing for Specific Blood Serum Parameters

About a quarter of TAR patients received ESAs as day 1 treatment, either alone or in combination with IV iron. Although a direct comparison is prevented by the reasons mentioned above, findings of the CAR and the ECAS reported a higher use of ESAs in Germany and Europe in 2001–2005 (CAR: planned ESA treatment rate of almost 40%; ECAS: approximately 45% of the patients were treated with epoetin, either alone or in combination with iron and/or transfusion) [2, 3]. Since then, safety concerns have led to revisions of existing practice guidelines [10, 12]. A summary of meta-analyses on ESA use in cancer patients from 2011 came to the conclusion that, overall, ESAs reduced the risk for RBCTx and increased the risk for thrombovascular events and mortality, while the effect of ESAs on mortality in patients receiving chemotherapy was unclear [22]. A Cochrane meta-analysis found no evidence for increased mortality in patients with target Hb <12 g/dL, undergoing chemotherapy [23]. Thus, in clinical practice, the benefits and risks of ESAs and transfusions should be carefully considered and decisions should be made based on each patient's situation and preferences.

**Figure 3:**
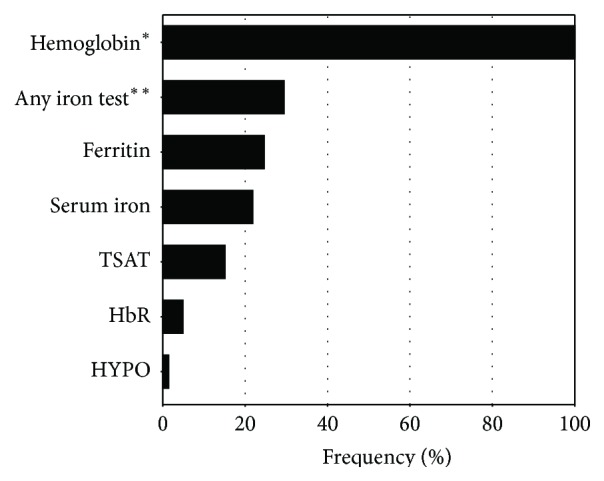
Frequency of patients tested for iron parameters at the start of antianemic treatment. ^*∗*^ Inclusion criterion; ^*∗∗*^ Patients tested for any of the listed iron parameters: ferritin, serum iron, TSAT (transferrin saturation), HbR (hemoglobin content of reticulocytes), or HYPO (hypochromic erythrocytes).

In this context, it is of great concern that only approximately one-third (30%) of all patients in TAR were tested for iron parameters at the start of antianemic treatment, most frequently by measuring ferritin, serum iron, or TSAT within 4 weeks before the start of therapy. Testing for Hb content of reticulocytes and hypochromic erythrocytes occurred even less frequently (Figure 3). Iron parameters were measured more often in patients with colorectal cancer than in patients affected by other malignancies (43% versus 28%, data on file).

Evaluation of nutritional deficiencies other than iron was rarely done; <1% of patients were analyzed for deficits in vitamin B12 and folic acid, respectively (data on file).

While it is possible that a proportion of patients had been tested prior to the four weeks before inclusion into TAR, this is unlikely to account for almost 70% of patients without documented diagnostics. Only 20% of patients had received antianemic treatment in the four weeks prior to inclusion. Data from the CAR reported that 44% of patients had been tested for ferritin and 33% for TSAT, although the time frame was not restricted and could have been more than four weeks before treatment [3].

While the NCCN recommends thorough diagnostics for possible treatable causes of anemia, including AID and FID, and specifies the parameters to be tested, no guideline on the diagnostics and treatment of cancer-related anemia has been published in Europe to date. In the TAR study, testing for iron parameters was performed more often in patients with colorectal cancer than in patients affected by other malignancies, accompanied by a higher frequency of IV iron therapy in this patient subgroup. This indicates that physicians currently use diagnostics for specific subsets of patients rather than as a routine requirement prior to any antianemic therapy.

IV iron has been shown to improve the efficacy of ESAs in patients with FID and is thus recommended for this patient subgroup [10, 12]. In addition, IV or oral iron is the treatment of choice in patients with AID [12]. In the TANDEM study, a diagnostic algorithm to select patients to antianemic treatment was suggested [24] based on the diagnostic plot by C. Thomas and L. Thomas [25] and identified about 25% of patients with iron deficiency in a cohort primarily designated for ESA treatment.

### 3.4. Effectiveness

Overall, antianemic treatment was successful in approximately half of all patients (“responders,” Table 2). Data on effectiveness are limited by the observational study design. There is considerable heterogeneity between the patients and thus effectiveness of treatments and QoL should not be compared between the different types of therapies. Causal relations cannot be drawn. Patient characteristics and inclusion criteria, such as baseline Hb levels <11 g/dL (females) or <12 g/dL (males), have to be considered when comparing data with other published studies.

Patients receiving transfusions had median final Hb between 1.2 and 1.6 g/dL above baseline, depending on the type of disease (ΔHb(final), Table 2). The maximum median increase after the start of treatment was between 2.6 and 2.9 g/dL (ΔHb(max), Table 2). Patients receiving ESAs showed median final Hb between 1.9 and 2.1 g/dL above baseline (ΔHb(final), Table 2). The maximum median increase was between 2.6 and 2.7 g/dL after the start of treatment (ΔHb(max), Table 2). Patients with inoperable solid tumors treated solely with IV iron, who in general had higher baseline Hb values (median 9.6 g/dL, Table 1), showed median final Hb of 1.1 g/dL above baseline. Due to the small number of patients, effectiveness parameters for other subgroups should be interpreted with caution.

The majority of patients receiving no RBCTx as antianemic therapy required no additional transfusions during the observation period (Table 2). According to the criteria defined in this study, all treatments were on average effective within the patient populations investigated. More than half of all patients showed a long-term rise in Hb levels. The main purpose of antianemic treatment is not only to correct Hb levels, but also to improve QoL [3].

### 3.5. Quality of Life

**Figure 4:**
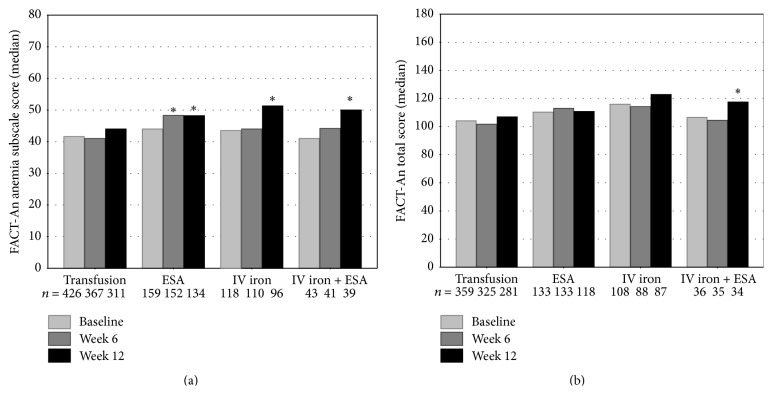
Quality of life (QoL) of patients assessed by the FACT-An questionnaire at baseline and after 6 and 12 weeks of the observation period. (a) Anemia-specific subscale score range [0–80]; ^*∗*^a difference of 4 points is considered clinically relevant [14, 15]; (b) FACT-An total score range [0–188]; ^*∗*^a difference of 7 points is considered clinically relevant [14, 15]; higher scores indicate a better QoL; numbers indicate the number of questionnaires returned.

While QoL is being measured more frequently in clinical trials, data on QoL in unselected, real-life patients are still rare. The FACT-An questionnaire is a validated tool to assess QoL in anemic cancer patients and to discriminate patients by their Hb levels and physical performance status [26]. In total, 78%, 70%, and 60% of the patients returned QoL questionnaires at baseline, after 6 weeks, and after 12 weeks, respectively. Median baseline FACT-An total scores (maximum 188 points) indicating overall QoL were between 104.1 and 115.9 points for all patients, with patients receiving transfusions having the lowest score (Figure 4(a)). Median baseline anemia-specific subscale scores (maximum 80 points) were <45 for all patients (transfusion: 41.6, ESA: 44.0, IV iron: 43.5, and ESA + IV iron: 41.0; Figure 4(b)). The median anemia-specific subscale scores showed improvement in all treatment groups. Clinically meaningful changes (≥4 points) were observed after 12 weeks for patients receiving ESA (44.0 to 48.2 points), IV iron (43.5 to 51.3 points), or ESA + IV iron (41.0 to 50.0 points). For patients receiving ESAs, clinically meaningful changes were already observed after 6 weeks (44.0 to 48.2 points). Overall QoL, as measured by the FACT-An total scores, also showed a median improvement after 12 weeks for patients receiving IV iron or ESA + IV iron. The difference reached the level of clinical relevance (≥7 points) for patients receiving ESA + IV iron (106.5 to 117.5 points); however, due to the small number of patients, this result should be interpreted with caution.

On average, a clinically meaningful improvement in the anemia-specific subscale scores was observed for TAR patients undergoing therapies other than RBCTx. However, this has to be interpreted with caution and might not be caused by the treatment applied, since patients receiving transfusions had lower Hb values at the start of treatment among other differences, which may also affect QoL. Improvement in QoL during antianemic treatment was also recently reported for patients receiving darbepoetin in German routine practice [7].

## 4. Conclusion

The aim of the TAR study was to assess the current treatment of anemia in cancer patients in German routine practice. Our data show that the majority of patients receive RBCTx, while ESAs, IV iron, or a combination of both is applied less frequently. Diagnostic testing for iron or other nutritional deficiencies is not routinely performed before treatment. All antianemic treatments were effective within the patient populations examined. Therefore, our data suggest that diagnostics for possible causes and causal therapies of anemia are underused in German routine practice. The large proportion of patients treated with transfusions, especially with Hb values ≥8 g/dL, highlights the need for systematic studies on the benefits of diagnostic-led treatment decision-making and for a European guideline on anemia management. It urgently needs to be discussed whether thorough diagnostics and earlier intervention can decrease the need for transfusions, at least in subsets of patients.

## Figures and Tables

**Table 1:** Characteristics of patients receiving antianemic treatments in German routine practice.

	Operable solid tumor (*n* = 207)					Inoperable solid tumor (*n* = 606)					Hematological malignancy (*n* = 149)					Total
Day 1 treatment	Transfusion	ESA	IV iron	ESA + IV iron		Transfusion	ESA	IV iron	ESA + IV iron		Transfusion	ESA	IV iron	ESA + IV iron		
Number of patients [*n*]	114	51	30	12		362	108	102	34		95	37	10	7		962
Sex																
Male [%]	36.8	27.5	40.0	50.0		50.0	43.5	52.9	58.8		57.9	51.4	80.0	57.1		48.0
Age at start of therapy																
Mean ± StD	66.0 ± 12.8	64.2 ± 12.5	66.2 ± 9.5	66.4 ± 8.8		66.6 ± 10.6	64.4 ± 9.7	66.8 ± 10.6	69.1 ± 9.2		69.2 ± 11.5	64.8 ± 17.0	67.7 ± 14.3	67.4 ± 10.4		66.5 ± 11.3
CCI^a^ [0–36]																
Mean ± StD	0.6 ± 1.6	0.5 ± 0.9	0.5 ± 0.9	0.8 ± 1.1		0.8 ± 1.3	0.6 ± 1.2	0.6 ± 1.1	0.6 ± 1.0		0.5 ± 0.9	0.8 ± 1.3	0.3 ± 0.7	1.4 ± 2.9		0.7 ± 1.2
Karnofsky Index [0–100]																
Mean ± StD	81.6 ± 11.5	82.2 ± 9.2	87.7 ± 8.2	86.7 ± 4.9		78.7 ± 12.8	82.5 ± 8.8	78.8 ± 12.0	80.6 ± 10.7		82.6 ± 12.5	82.2 ± 10.0	77.0 ± 14.9	88.6 ± 6.9		80.7 ± 118
Most frequent solid cancers [%]																
Breast	16.7	41.2	16.7	8.3		14.1	25.0	11.8	14.7		—	—	—	—		14.7
Colorectal	15.8	13.7	30.0	33.3		9.1	5.6	20.6	2.9		—	—	—	—		10.3
Lung (NSCLC)	10.5	11.8	3.3	16.7		10.8	15.7	3.9	5.9		—	—	—	—		8.6
Tumor therapy [%]																
No therapy	6.1	—	3.3	—		2.5	0.9	2.0	—		6.3	5.4	20.0	—		3.1
Chemotherapy	92.1	98.0	93.3	100.0		88.1	95.4	86.3	94.1		76.8	81.1	40.0	85.7		88.4
Other	1.8	2.0	3.4	—		90.6	96.3	88.3	5.9		16.9	13.5	40.0	14.3		91.5
Baseline Hb																
Median [g/dL]	8.7	9.3	9.6	9.6		8.6	9.5	9.6	9.4		8.5	9.2	9.6	10.8		8.9

^a^Charlson Comorbidity Index.

**Table 2:** Effectiveness of antianemic treatments in German routine practice.

	Operable solid tumor (*n* = 207)					Inoperable solid tumor (*n* = 606)					Hematological malignancy (*n* = 149)			
Day 1 treatment	Transfusion	ESA	IV iron	ESA + IV iron		Transfusion	ESA	IV iron	ESA + IV iron		Transfusion	ESA	IV iron	ESA + IV iron
ΔHb(max)^b^ [g/dL]														
*n* ^a^	78	41	18	11		257	79	71	27		67	34	7	5
Median	2.9	2.7	2.0	2.6		2.7	2.6	2.0	2.3		2.6	2.6	2.0	2.9
Mean ± StD	3.1 ± 1.5	3.0 ± 1.4	2.3 ± 1.2	2.8 ± 1.3		2.8 ± 1.8	2.8 ± 1.5	2.0 ± 1.6	2.5 ± 1.3		2.6 ± 1.5	2.5 ± 1.9	2.6 ± 1.5	2.8 ± 1.5
ΔHb(final)^c^ [g/dL]														
*n* ^a^	63	40	16	11		223	77	67	26		62	32	7	5
Median	1.6	2.0	1.6	1.7		1.5	1.9	1.1	1.8		1.2	2.1	2.0	2.3
Mean ± StD	2.0 ± 1.8	2.1 ± 1.2	1.7 ± 1.5	2.1 ± 1.3		1.6 ± 1.5	2.1 ± 1.7	1.3 ± 1.5	1.9 ± 1.5		1.6 ± 1.8	1.9 ± 2.0	2.4 ± 1.6	2.2 ± 1.9
Responders^d^														
*n* ^a^	63	40	16	11		223	77	67	26		62	32	7	5
%	50.8	72.5	62.5	63.6		41.3	55.8	43.3	57.7		35.5	59.4	57.1	80.0
Transfusions														
*n* ^a^	114	51	30	12		362	108	102	34		95	37	10	7
Weeks 1–4 [%]	100.0	25.5	13.3	8.3		100.0	26.9	16.7	17.6		100.0	29.7	10.0	—
Weeks 5–8 [%]	24.6	5.9	6.7	—		31.5	14.8	9.8	2.9		44.2	10.8	10.0	14.3
Weeks 9–12 [%]	17.5	3.9	3.3	—		26.2	8.3	7.8	5.9		33.7	21.6	20.0	—

^a^Number of patients for whom variable is documented or could be calculated.

^b^The maximal difference between the baseline Hb and the highest Hb documented.

^c^The difference between the baseline Hb and the last Hb documented within the 12-week observation period, but at least 4 weeks after the start of treatment.

^d^A responder is defined as a patient with final Hb > 11 g/dL or with ΔHb(final) of ≥ 1.5 g/dL, with final Hb being the last documented Hb within the observation period, but at least 4 weeks after the start of antianemic treatment.
